# UAV-Based Photogrammetry and Integrated Technologies for Architectural Applications—Methodological Strategies for the After-Quake Survey of Vertical Structures in Mantua (Italy)

**DOI:** 10.3390/s150715520

**Published:** 2015-06-30

**Authors:** Cristiana Achille, Andrea Adami, Silvia Chiarini, Stefano Cremonesi, Francesco Fassi, Luigi Fregonese, Laura Taffurelli

**Affiliations:** Department of Architecture, Built Environment and Construction Engineering, ABC, Politecnico di Milano, via Ponzio, Milano 31-20133, Italy; E-Mails: cristiana.achille@polimi.it (C.A.); andrea.adami@polimi.it (A.A.); silvia.chiarini@polimi.it (S.C.); stefano.cremonesi@polimi.it (S.C.); francesco.fassi@polimi.it (F.F.); laura.taffurelli@polimi.it (L.T.)

**Keywords:** tall buildings, close range photogrammetry, UAV application, automated 3D modeling techniques, terrestrial laser scanning, topographic survey, cultural heritage, emergency context survey

## Abstract

This paper examines the survey of tall buildings in an emergency context like in the case of post-seismic events. The after-earthquake survey has to guarantee time-savings, high precision and security during the operational stages. The main goal is to optimize the application of methodologies based on acquisition and automatic elaborations of photogrammetric data even with the use of Unmanned Aerial Vehicle (UAV) systems in order to provide fast and low cost operations. The suggested methods integrate new technologies with commonly used technologies like TLS and topographic acquisition. The value of the photogrammetric application is demonstrated by a test case, based on the comparison of acquisition, calibration and 3D modeling results in case of use of a laser scanner, metric camera and amateur reflex camera. The test would help us to demonstrate the efficiency of image based methods in the acquisition of complex architecture. The case study is Santa Barbara Bell tower in Mantua. The applied survey solution allows a complete 3D database of the complex architectural structure to be obtained for the extraction of all the information needed for significant intervention. This demonstrates the applicability of the photogrammetry using UAV for the survey of vertical structures, complex buildings and difficult accessible architectural parts, providing high precision results.

## 1. Introduction

Which kind of surveying strategies should be chosen in the case of complex and critical situations? This paper explains how traditional and innovative techniques in the surveying field can be applied to obtain high quality timely results in complex contexts. This case study deals with for tall buildings and critical situations like in the case of the earthquake that affected two Italian regions, the Emilia and Lombardy regions, in May 2012.

A great number of historical buildings were seriously damaged by the shocks. In particular, most of the churches located in the southern area of Mantua’s province required very important structural interventions. In the city, recently declared a UNESCO World Heritage (2008) site, the Santa Barbara bell tower was heavily impacted by the effects of the earthquake and shows many problems.

To repair the damages, the first in-depth fact-finding operation is an accurate survey of the architecture and its structure. To acquire and describe complex architectural objects, researchers required a 3D database of spatial and metric information that can be used to extract three-dimensional models as well as two-dimensional representations and all the information needed for the maintenance program or restoration projects [1,2,3,4,5].

The bell tower of Santa Barbara has, like every tower, a structure that is quite challenging to measure correctly due to its natural vertical extension. Moreover, the dense urban pattern of the old town center makes it hard to find good positions to observe and measure all four facades from a horizontal point of view. The possible acquisition points are on the ground, very close to the tower and this creates bad measuring angles, a non-uniform resolution on the object, noisy scans, and consequently low accuracy measurements. Moreover, in this case the lantern at the top of tower cannot be seen from the ground floor, making the use of lifting equipment indispensable. An additional problem to be resolved is the presence of temporary holding structures around the top lantern that hide the structure and impose multi-angle acquisition from a close point of view.

In light of all these problems, the most suitable solution for the 3D survey of the bell tower is the integration of different sensors and methods in order the use the potential of every method correctly [6,7,8]. In particular, the idea for the external facades was to use a manually controlled UAV.

The solution for the 3D modeling of tall structures is the integration of laser scanner data of the internal areas with dense image matching DSM of the external façades, using a classical topographic network to georeference all data together in a single reference system. Some aspects have to be considered in order to plan the different acquisition correctly. The integration of different data requires sufficient overlap areas, same resolution and comparable accuracy.

After an opening overview on the specific architecture and its damage after the earthquake, a first analysis in this paper regards the traditional methods for the survey of tall buildings and towers. Particular attention is given to the most recent techniques of automated 3D modeling and UAV acquisition in order to highlight their pertinence in the survey of Cultural Heritage artefacts. Before describing the survey stage, some tests to verify the possibility of integration of automated image modeling with laser scanner data are described.

## 2. Santa Barbara Tower Bell and the Earthquake

**Figure 1:**
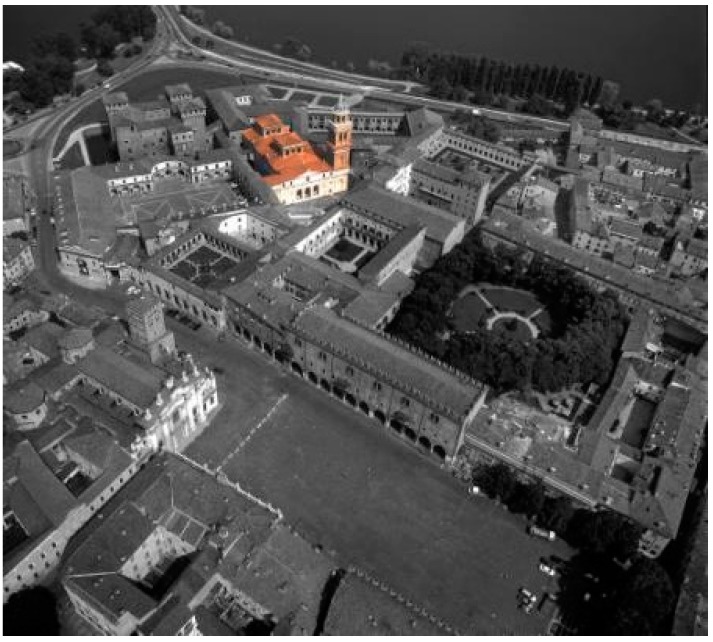
Santa Barbara Church and its bell tower in the historical urban context.

The church of Santa Barbara is located in the old town center of Mantua and is one of the most representative buildings in the city (Figure 1).

Santa Barbara church was commissioned by Guglielmo Gonzaga as the family chapel inside the “Palazzo Ducale”, the most important historical complex of Mantua. The architect Giovanni Battista Bertani built it in 1562–1572. The bell tower, with a height of about 49 m from the top of the dome, has a square plan (about 9.00 × 9.00 m). It is composed of six internal levels. Some ledges split the main tower in three parts; the first one is finished with plaster and the others have a brick surface. The facades have different architectural elements like niches, gables, arches and pilaster strips. In the belfry, two arches on every façade show the bells to the exterior. Above this point, there is a small round temple with a colonnade, covered by a balcony and a little dome with a lantern: this is the most characteristic part of the bell tower that characterizes the Mantua landscape.

**Figure 2:**
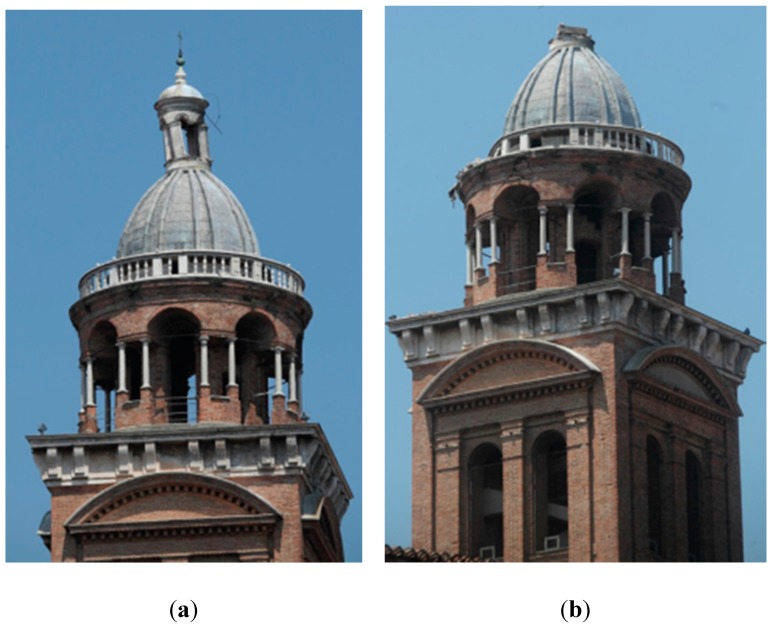
Santa Barbara bell tower, after the first earthquake (**a**) and the second (**b**) with the collapse of the lantern.

On May 20th and 29th 2012 two big earthquake shocks were felt across a large part of the north of Italy. During that earthquake many buildings were damaged (Figure 2) and afterward it was necessary to batten down the hatches by building temporary structures for safety of the buildings and people.

In the Santa Barbara bell tower, the top was the most damaged part. The dome’s lantern collapsed and in its fall broke off a part of the summit balcony, in addition to some areas of surrounding buildings. The round temple was subjected to a rotation movement and the structure had some subsidence, so many fissures appeared to the structure.

The serious damage that affected the highest part of the structure required the immediate realization of a temporary holding structure. In the initial days after the collapse, the “Protezione Civile” fire brigade secured the top of the tower with steel cables and a reticular tubular structure to prevent another collapse, before any detailed survey could be done.

The project of the structure for the support of damaged parts, to save time, was based on a drawing made in 1999, during the last restoration work, and some photographs taken using a large crane. Because of the lack of complete and up-to-date drawings, the Cultural Heritage office of Mantua’s Diocese asked for all the representations needed for the analysis of the present situation (e.g., material decay, tower’s tilt and structural damages) and to design the restoration and complete the bell tower. Therefore, it was considered necessary to make an accurate survey of the situation after the earthquake to obtain traditional two-dimensional architectural representations (plans, elevations and sections) together with the addition of vertical profiles, facades, orthophotos and a 3D model for finite element calculation. The survey was intended at an overall scale 1:50, which requires an accuracy of less than 1 cm, with parts detailed at 1:20 scale.

## 3. Traditional Approach

The main problem for the operational stage of the survey was the acquisition of the highest part of the tower, due to its height, and the Northeast facade, which is set against the church’s sacristy. In particular, the belfry and the round temple cannot be measured from the ground. Moreover, it was necessary to guarantee a high precision result to allow a correct and detailed design of the new structure. Before starting the survey of the bell tower, many possible solutions were considered based on traditional or well-established methods of architectural survey: topography, laser scanning and traditional digital photogrammetry.

The topographic survey is surely one of the most well-established methods. It allows all the significant points of the facades of the tower to be acquired. It requires many station points, adjusted in a topographical network, including acquiring the measurements via the method of irradiation or, better, forward intersection [9]. All this requires the possibility of moving around the tower and, the possibility of identifying raised measuring positions. In addition to the logistic difficulties, which are different case by case, the critical aspects of this method are related to time. The operation of discretization, to determine which points and parts of the architecture have to be represented, cannot be referred to the next stage of the restitution, but it must be done directly on site. This requires lengthy survey times, high logistic difficulties and moreover this methodology can no longer be used with the arrival of methods that provides high resolution measurements directly in 3D, such as laser scanner or photogrammetry, in real time or nearly real time. Topography or single point measurements methods are however necessary to register different data together and to test the results.

The photogrammetric survey shortens the acquisition time on site, as most of the information is extracted in post-processing. The capture geometry is the challenge of the photogrammetric acquisition. Acquiring images with the correct geometry is extremely necessary in order to achieve the requested precision that means, in this case, finding some suitable high places, such us balconies or roofs of surrounding buildings. Images, taken with very narrow angles, make the restitution phase more complex and, in any event, provide measurements of lower accuracy.

For laser scanning technology, in addition to the foregoing consideration, it is also necessary to analyze the different kind of instruments. Some instruments, especially the ones based on phase shift technology, have a short measurement range. This means that when the scan is made from the base of the tower or from other places around it, acquired data can be very noisy. From relevant distances, moreover, the point clouds do not have a high resolution and therefore the result cannot be very accurate.

## 4. The Most Recent Surveying System

The latest surveying systems deal with both the acquisition stage and the data processing. One of the most important changes in recent years regards the combination of digital photogrammetry and computer vision into new software for automated 3D modeling from images. A second innovation concerns the possibility of acquiring information (images, point clouds, IR images) from above, with remotely piloted aerial vehicles.

The integration of these two latest methods—automated 3D modeling from UAV acquired images—can solve the problems mentioned before. In fact, it is possible to obtain high resolution dense point clouds of tall buildings in a short time, even if the building is located in a high-density area.

### 4.1. Automated 3D Modeling

It is complex to find a single definition for this new approach to 3D modeling from images. By focusing the attention on different steps of the workflow, many definitions have been suggested: dense stereo matching (to underline the matching phase from stereo pairs), dense image matching (image as source of 3D), automated 3D image modeling (automatic approach from images).

However, this software appeared about 4 or 5 years ago and is the result of developments in the field of photogrammetry and computer vision. The most important innovation regarded the image matching process. It can be defined as the establishment of correspondences between images. The first studies in photogrammetry mainly concerned aerial images and topographic mapping problems. To foster image matching, the concepts of epipolar geometry and cross-correlation for image matching were introduced. However, it is only with the advent of digital images that research focused on the automation of procedures in order to avoid the intervention of skilled operators. Computer vision, on the other hand, focused on stereo matching but with a different aim, which was the reconstruction of 3D space, without particular attention to its accuracy. A second innovation was dealing with the possibility of automatically extracting dense point clouds from images by the use of some operators, which allows detecting and describing local features in images.

The meeting of the two sciences led to the three-dimensional reconstruction of reality, starting from images. Even with specific algorithms [10,11,12,13,14], these software allow the fully autonomous three-dimensional reconstruction of objects from images in different steps:

(a) Automatic extraction of characteristic features of images;
(b) Image matching to detect tie points;
(c) Computing by bundle adjustment of interior and exterior orientation;
(d) Computing of a dense point cloud.

In addition:

(e) Triangulation of points in a mesh;
(f) Texture mapping.

The ability to reconstruct three-dimensional objects from images is a common task in the Cultural Heritage field. Consequently, the possibility of using low cost systems (a simple digital camera) rather than expensive systems (laser scanners) was a relevant step. Additionally, these systems provide not only the geometry of the object, but also its texture that is metrically projected onto the 3D images. From this extracting orthophotos that are useful and widely used in this type of application is quite automatic and immediate. In addition, these determined its use not only in the surveying field, but also in virtual reconstruction or simulation. Ease of use, manageability and speed contribute to their spread. In addition, many algorithms have been developed around these methods to optimize the calculations and obtain accurate results with short time of elaboration. Several open source software packages have fostered the 3D reconstruction from images by providing an inexpensive solution and by allowing an in-depth analysis of results and algorithms.

Many researches were focused on the comparison between points-clouds from laser scanning and automated-photogrammetry [10,13,15]. Instead, one of the topics of this paper is to verify the accuracy of the method and the possibility of integrating all the data into a single reference system.

### 4.2. UAV Survey

The term Unmanned Aerial Vehicle (UAV), also commonly known as drones, defines a generic aircraft designed to operate with no human pilot onboard [16]. This acronym is commonly used in geomatics applications, but many other terms identify these aircraft according to their propulsion system, autonomy, maximum altitude and level of automation, giving an idea of the variety of the jobs carried out by these systems. For example drone, Remotely Operated Aircraft (ROA), Unmanned Combat Air Vehicle (UCAV), Medium Altitude Long Endurance (MALE) UAV, Remote Piloted Aircraft System (RPAS), *etc.* Another definition describing these devices is Unmanned Aerial Systems (UAS) that comprehends the whole system, including the aerial vehicle and the ground control station.

The development of UAV solutions was primary driven by military purposes, for inspection, surveillance, and mapping of inimical areas. The quick development of civilian solutions, in particular for geomatics applications, was made possible by the spread and improvement of digital camera devices and GNSS systems, necessary for navigation and geo-referencing tasks [17].

In geomatics, the use of UAVs is a low cost alternative to traditional airborne photogrammetry for large scale topographic mapping or recording detailed 3D information of terrestrial objects and integratation with the data collected by terrestrial acquisition (for example laser scanning). UAV aerial photogrammetry cannot replace traditional photogrammetry and satellite mapping application for large territories, but they give an efficient solution for little areas and large scale surveys. This is the case of Cultural Heritage applications. Moreover, these little flying vehicles give rapid solutions to some long-standing unsolved problems, such as for example the high resolution survey of pavements and vertical surfaces such as, for example, tall towers.

The principal frames for the UAV used for geomaticsphotogrammetry applications are unpowered (balloon, kite, glider), fixed wings, copter and multi-copter platforms. Each of them have different advantages and handicaps referring to different tasks and applications. There is also a wide price range, approximately from 1000 to 50,000 Euros depending on the on-board instrumentation, flight autonomy, payload and automation capabilities. For example, low cost solutions do not allow a completely autonomous flight, and in most cases, they require human assistance for take-off and landing. Another cost-sensitive element is the engine type: in comparison with the most used electrical ones, internal combustion engines have longer endurance and permit higher payloads, but they also have higher costs and require more maintenance and pre-flight controls. The three principal frameworks of drones, used mostly for geomatics purposes, have specific characteristics:

*Fixed wing*: these vehicles work like a traditional aircraft with one or more propellers and fly in a straight direction. Most of these drones work in a fully autonomous mode following a pre-planned flight plan. The characteristics of this structure made the drones suitable for aero-photogrammetry works due to high autonomy and possibility of cover wider areas than a multi or single rotor configuration. The most used and portable solutions are built with light materials (less than 1 kg take-off weight) for increased portability and autonomy. In contrast, this solution has very low resistance to wind—this makes it difficult to operate in high wind conditions, and limits the payload.

*Copters*: these devices are basically scaled-down models of real helicopters; usually the bigger ones are equipped with gasoline engines that give very high autonomy and the possibility of carrying a much heavier payload. This type of UAV has high flexibility. On the other hand, big copter systems are also characterized by a high resistance and stability in difficult wind conditions. A problem of the systems with ICE motors is the greater need for maintenance and preparation procedures for flying.

*Multi-copters*: they consist of devices with three or more propellers fixed to a structure. Position and motion are controlled by managing the differential engine rotation speed of any single propeller. This type of system is very flexible in relation to the various different tasks and payloads (more propellers means more payload).

Typically, the batteries do not have a very long autonomy (15–20 min approx.) meaning batteries must be changed for long sessions of flightwork. These systems are preferred very often for the application of terrestrial photogrammetric Cultural Heritage surveys, infrastructures and civil engineering because of their high maneuverability, small dimensions, short deployment time and high stability.

The development of these systems is very fast and it is focused on increasing the flight time, accuracy of on-board navigation systems and in particular on the sensors that can be used.

## 5. Laboratory Tests on Data Integration

Before integrating laser scanners and topographic surveys with photogrammetric ones, made by automated 3D image modeling, it was necessary to evaluate several aspects. In particular, regarding point clouds, density of points, quality and presence of color information had to be considered.

The first aspect was strictly related to resolution, i.e., the average density of points on the surveyed surface. A homogeneous density allows the same level of detail for each part of the object to be obtained, both in photographic representations such as orthophotos and in three-dimensional models.

Even more important is the quality of measurements, which involves the concepts of accuracy and precision. The first one expresses the ability to approach the real measurement, the second the ability of repeating the measurements and obtaining a similar value. The possibility of recording RGB values is an added value as it provides more information about the object.

The first condition to integrate different data sources is that all data must respect some pre-established parameters. The immediate example regards accuracy. The scale of the survey and complexity of the object in fact determine the minimum imposed value of accuracy. If one of the two datasets does not guarantee that minimum value, they cannot be integrated. Once this first condition is guaranteed, it is necessary that the values of accuracy and precision are very similar, otherwise, the errors in the data recording stage can produce problems in the calculation of the final products.

To verify the integrability of the two different techniques, laser scanner and digital automated photogrammetry, some tests were done to verify the quality of their data. The same object was surveyed with both methods and then the results were compared. In order to have a comparison that is closer related to the case of Santa Barbara bell tower, the test involved the base section of the tower. In that position both the laser scanner and photogrammetry had no logistic problems. Some targets, for photogrammetry or laser scanner, were measured by a total station (Leica TS30, Leica Geosystems, Italy) in order to verify the registration stage of different data and to evaluate the results in a single reference system. They were located on different levels of the facade in order to have a fully 3D spatial distribution of targets. Different datasets were acquired to make a complete comparison and they included images taken with reflex cameras and laser scanner scans. In particular:

- Dataset 1: Photogrammetry—Canon 5D
  The same area was surveyed by 41 images acquired by Canon EOS 5D Mark III (Canon, Italy) with a 35 mm lens. The EOS 5D is a high-resolution camera, but it has no calibration certificate. The images were shot in jpg format and their dimensions are 5760 × 3840 pixels. The pixel size is 0.0064 m and the ground sample distance (GSD) is 0.0016 m.
- Dataset 2: Laser scanner—HDS 7000
  Single point cloud made by a Leica HDS 7000 (the same instrument used for acquisition in the after-quake context in the Santa Barbara case) overhead at a distance of 8 m from the surface of the object. It has a linearity error, as defined by the producer, ≤ 1 mm. The scan is made up of 123 million points.

The laser scanner point cloud was considered as the term of reference. From the Leica datasheet, the HDS 7000 noise standard deviation is about 0.4 mm at 10 meters distance, in the event of grey objects. The point cloud was referenced in the topographic reference system by using four black/white targets (automatically extracted in Cyclone), with an average error of 2 mm. The other eight coded targets were picked manually and used as check points. The difference between the coded targets acquired by total station and manually picked in the oriented point cloud is very small: the average error is 0.001 meter. The photogrammetric dataset was processed in Agisoft Photoscan [18] to verify their integrality with laser scanner point clouds and to verify the quality of automated 3D image modeling. The images were aligned and then were georeferenced by using coded targets, automatically detected by the software. Some coded targets were not used for registration as GCP, but as checkpoint (CP) for accuracy verification. For an appropriate analysis work, all data were elaborated in different ways, e.g.,

Test 1 automatic image-based modeling without targets
Test 2 automatic image-based modeling with topographic targets
Test 3 automatic image-based modeling with topographic targets, optimised.

In Table 1 it is evident that by using acquired targets and the optimisation process the errors are adjusted.

**Table 1:** Comparison of dataset orientation.

	*n* Photos	Optim.	RMSE [Pixel]	GCP	CP	Mean GCP with TCRA [m]	σ_xyz_ GCP with TCRA [m]	Mean CP with TCRA [m]	σ_xyz_ CP with TCRA [m]
Test 1	41	no							
Test 2	41	no	0.145	9	4	0.000925	0.000480	0.000740	0.000203
Test 3	41	yes	0.145	9	4	0.000876	0.000415	0.001012	0.000292

Moreover, the level of accuracy achieved is very high. It means that such data, obtained by automated image modeling, are surely suitable for an architectural survey at 1:50 scale and also 1:20. By considering the accuracy level of laser scanner point cloud, it is evident that photogrammetric data can be fully integrated with the laser scanner data.

A further comparison regarded not the result of georeferencing, but the final results of the processing stage. By means of an analysis based on the Euclidean distance, the point clouds, obtained from two datasets, were compared. It was decided to carry out a comparison of point clouds because they are the first result of laser scanning and photogrammetric methods. Furthermore, this choice allowed any errors or systematic effects due to triangulation algorithms to be excluded.

**Figure 3:**
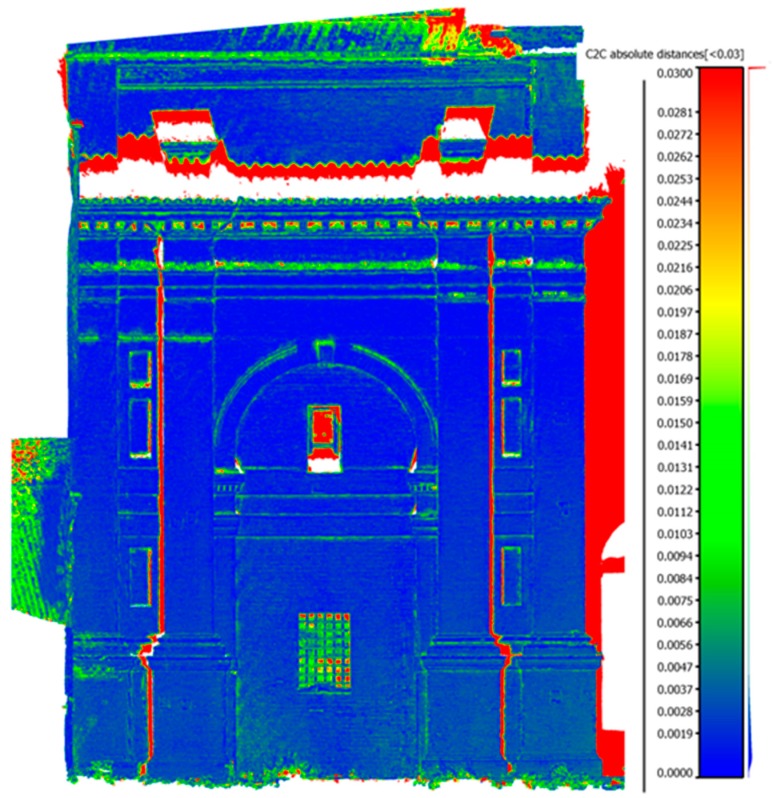
Comparison of photogrammetric point clouds with laser scanner one. The maximum difference, shown in red color, is 3 cm and it is present only on the borders or in missing parts.

The comparison was made in Cloudcompare [19], an open source software designed for data verification between point clouds or meshes. The point cloud of the Canon dataset, geo-referenced in the same reference system of topography and laser scanner, was compared with the laser scanner one. The differences, as shown in Figure 3, are very small and are mainly concentrated on the edges where the laser scanner data is less reliable. This test verifies that the point clouds produced for photogrammetry, both semi-metric and amateur camera, are very similar with regards to accuracy and precision and they can be integrated into a single system. In such a way, it is possible to survey complex objects with different methods that can be finally integrated to create, for example, a single 3D database.

## 6. The Survey of the Santa Barbara Church Bell Tower

As described before, in the survey of the bell tower all techniques, both traditional and innovative, were applied in order to obtain the best results and to solve the problem in the documentation of different parts. The acquisition of the interiors of the tower and the lowest exterior parts did not involve any kind of problem and the survey was conducted in a traditional way. The most complex part was, as expected, the upper part and in particular the exterior of the belfry and the round temple*.*

The first step was the realization and materialization of the topographic network to define the reference system for all the work by using the TS30 Leica Total station. The points of the network were evenly located around the tower, outside (n = 5 points), and at different levels, inside the tower (n = 16 points). From those stations 95 targets (GCP) where acquired to georeference scans and images. For the highest part of the tower, an additional ground station was created on a balcony in front of the bell tower and connected with the main topographic net on the ground.

Laser scanner acquisition was carried out with a Leica HDS 7000. It is a phase shift instrument with an onboard control system. It is a class 1 laser product with an acquisition range between 0.3 and 187 m and a 360 × 320° field of view. This instrument can acquire up to 1 million points/second at the maximum resolution. The final precision depends on several causes like instrument features, object distance from the instrument, surfaces materials, and angle of incidence of laser beam to the surfaces, *etc.* [20,21,22].

**Figure 4:**
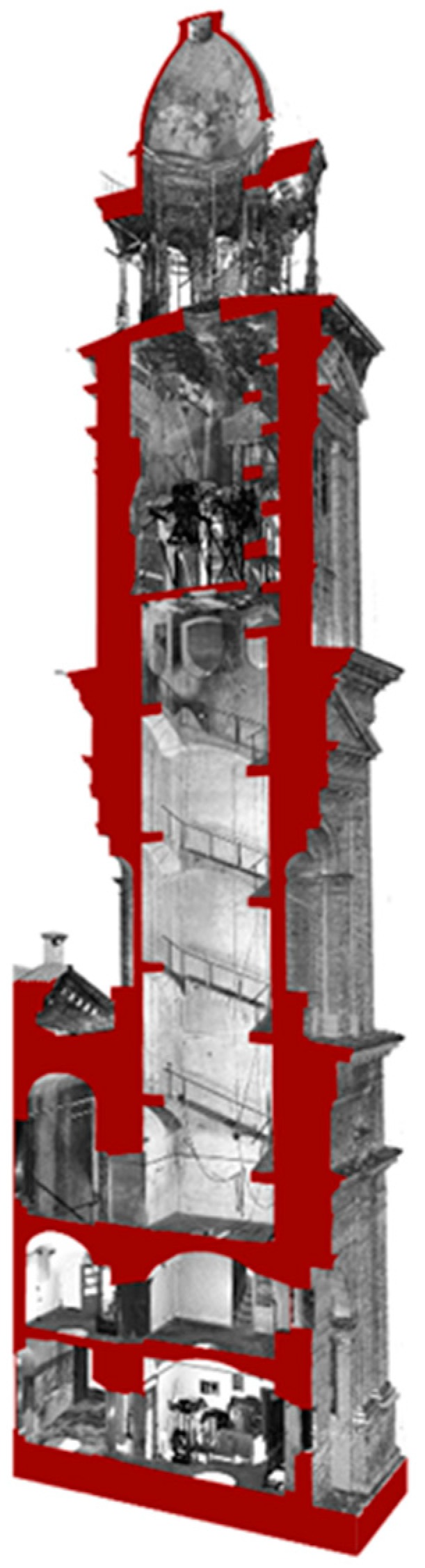
Laser scanner point clouds of interior and exterior registered in the local reference system for the elaboration of the section.

Many scans were necessary to acquire the total bell tower: 15 for the exterior (nine from the ground floor and six from balconies on the nearby buildings), 23 for the interior (scale, sacristy, and bells zone) and 12 for the lantern acquisition (in Figure 4 a section of the bell tower extracted from interior and exterior scans).

The resolutions of the interior and exterior surfaces are quite different. The interior surfaces had a more uniform resolution (3 mm at a distance of 4.5 m). Instead, for the façades, the resolution is always less, rising as the scans were done from the base of the tower, with a fixed angular step (the greater the distance, the lower the resolution). There is another effect to consider due to the height, namely the accuracy. In fact, the laser beam hit the surfaces with a very low, higher incidence angle and a bigger laser beam, resulting in a decrease of accuracy.

The first solution, trying to produce a high defined and complete acquisition of the top of the tower, was to position the laser scanner on the corner of the highest ledge, around the round temple, for the acquisition of the structure comprising arches and columns, and on the highest balcony for the acquisition of the little dome.

**Figure 5:**
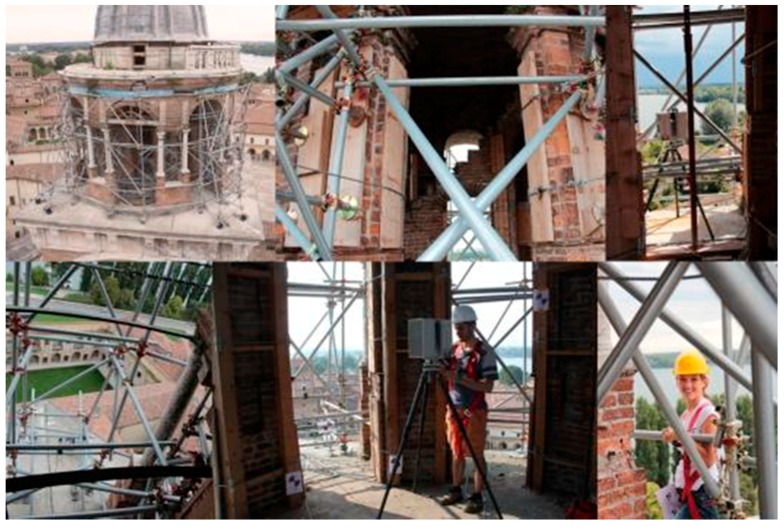
Some pictures of survey moments of the “round temple”, on the top of the tower. The images shows the difficulties given by the presence of the metal scaffoldings and the wooden holding structure positioned after the earthquake.

In this case, the main operational issue for the acquisition of a clear data was the small space surrounding the round temple. It is located at a height of 38 m from the ground, it has a diameter of about 8 m and it is placed on a 10 m square plan, which has no railing. The main problem was the presence of the temporary metal and wooden structures, placed after the earthquake, that still hold the round temple*,* preventing further collapses and occupying a large part of the space (Figure 5). These structures, mostly comprising metal pipes, made transportation of the laser scanner instrument to the top of the tower of the tower and its placement difficult. They also disturbed the acquisition of the architecture by reflecting the laser beam, moreover, in the test scans acquired by the extreme corner of the ledge the pipes obstructed the view of several architectural parts. The high balcony was not accessible because of the instability of the round temple stairway and the wooden supporting structure that was obstructing the only access door.

### 6.1. Image Acquisition of the Top of the Bell Tower

**Figure 6:**
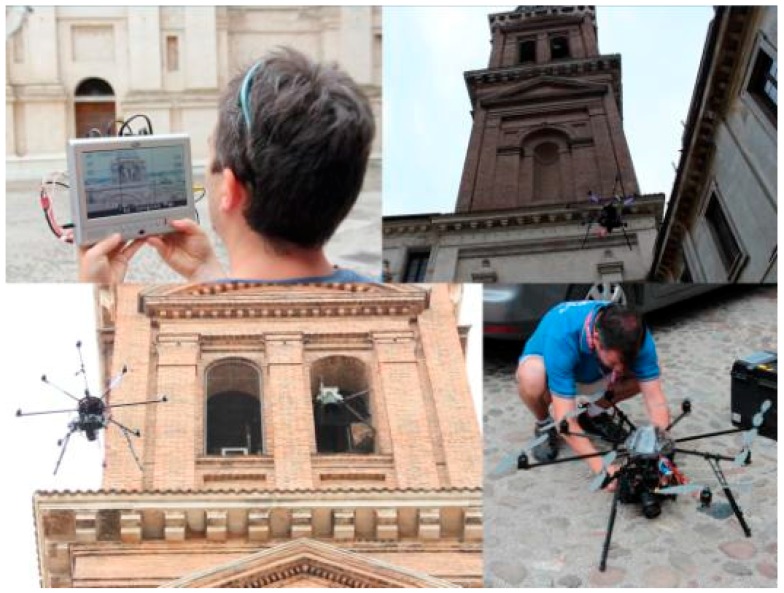
The octocopter employed for the bell tower acquisition, and the Wi-Fi camera controller.

One way to complete the survey of the tower was to use an UAV system. This avoided the use of cranes, which would lead to higher costs and time expenditures. Moreover, the possibility of moving a simple crane would not allow the acquisition of vertical strips without the use of *ad hoc* devices, and it would have been impossible to reach all the acquisition positions needed. Therefore the acquisition was performed in collaboration with Eos Fly (Mantua, Italy) using an octocopter (Figure 6).

The choice to use a multi-copter was made by taking different aspects into account. The first consideration was the type of building: a vertical and very tall structure. UAV allows a vertical flight pattern so it permits the acquisition of vertical strips of images. Another consideration was based on the position of the building, which is in the old town center of the city, surrounded by other buildings. For this reason it was necessary to use an easy to handle vehicle.

The flight device had eight propellers fixed on the same number of arms, two gyroscopes for the flight control and the telemetry instruments (GPS and the barometric altimeter). The octocopter had a flight autonomy of about five to fifteen minutes, depending on the weight loaded on board; it was equipped with LiPo batteries (16 V 4.0 Ah). The octocopter was equipped with a reflex camera (Canon EOS 650D, APS, 18 Mpixel), the camera mount could tilt 90° vertically, from horizontal position to a zenithal one. The RC system controls both the fly operations, the camera rotation and camera trigger. The flying team included the pilot and by a photogrammetric expert able to visualize the camera view on a remotely connected monitor. This was the way to acquire images with the correct point of view and overlap.

The most relevant step was the flight plan. It is important to define the distance from the surface, the overlap between images and, as a consequence, the trajectory. To optimize the acquisition time and reduce the number of photos, the project was optimized taking into account the camera parameters, dimension and characteristics of the building and the surroundings.

The employed camera was a Canon EOS 650D with a CMOS sensor size of 5184 × 3456 pixels (22.3 × 14.9 mm) and 18 mm focal length lens. Each image was acquired with an aperture f/9 and 400 ISO. A maximum pixel size (GSD) on the object of about 3 mm was calculated, which involves an average distance of about 8 meters from the surface. An overlap of about 80% between neighboring images was expected.

**Figure 7:**
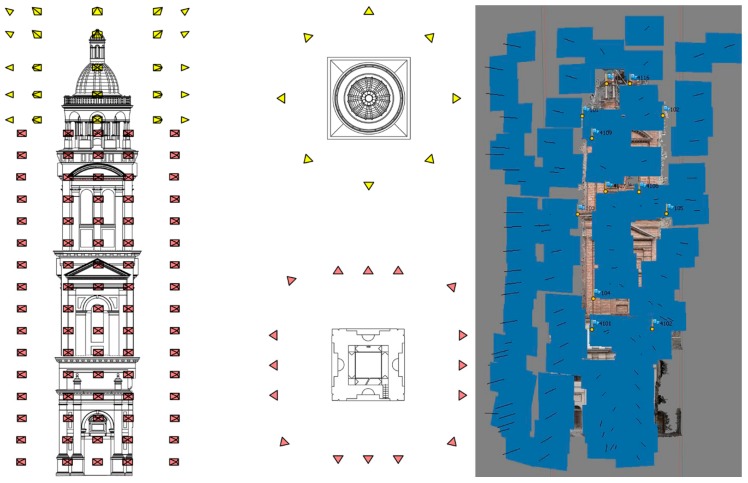
Design of UAV image acquisition. In red the images for the front of the bell tower, in yellow for the round temple. Result of image orientation. The difference between project and real flight are evident.

The plan (Figure 7) was to acquire three vertical image-strips for each front, completed by two additional strips on the corners, which would permit the connection between adjacent fronts. For the acquisition of the round temple it was planned to realize three 360° flights around it, with a minimum of eight shots, completed with the same number of oblique shots from highest positions and a series of nadir photos.

During the acquisition phase, it is recommended to have the same light conditions in order to have uniform color and illumination in each image. At the same time, shadows should be avoided. In this way, the photogrammetry texture and the orthophoto are uniform and similar in every part of the structure. For this reason, an overcast day was chosen to survey Santa Barbara allowing optimal light conditions.

Overall, 110 photos were taken for the north facade, 19 for the south one, 70 for the east, 83 for the west one and 159 for the round temple. The different numbers of images was due to the different size and position of each façade and the presence of structures. A 77% overlap was obtained with just a vertical baseline of 2.35 m and a horizontal one of 3.48 m. Only the middle strip revealed enough to acquire over the 90% of the facade width, consequently each point on the object’s surface was recognized at least in eight photos (Figure 7).

### 6.2. Data Processing

The laser scanner and topographic surveys were completed in three days of work. As is known in survey operations, the processing phase is longer than acquisition due to the amount of data to process and the manual processing of features extraction: the only laser scanner database comprised 2.3 billion points. For the complete representation of the bell tower, the strategy was to use both a laserscanner and photogrammetric pointclouds. Photogrammetric data were integrated with laser scanner ones for the orthophotos of the lower external parts of the facades, whereas the interiors were drawn only from laser scanner point clouds.

The photogrammetric elaboration was done using Agisoft Photoscan. Due to the high number of images, it was more advisable to divide the project into sub-parts (“chunk” in Photoscan). This allows the elaboration time to be reduced, splitting it onto different PCs. The bell tower exterior model was subdivided into different parts corresponding to every single façade. Overall, five chunks were created, one for the round temple and one for each side.

**Figure 8:**
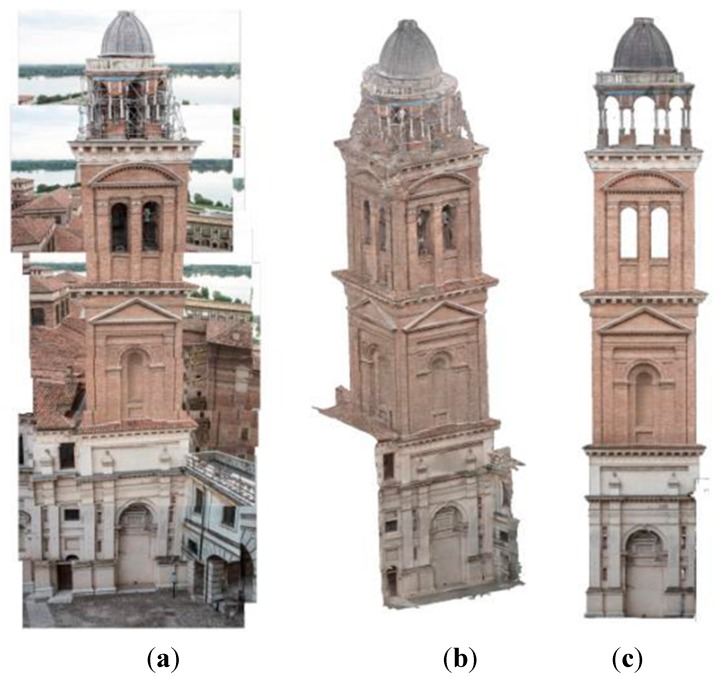
(**a**) example of vertical strip acquisition; (**b**) image-based model; (**c**) front orthophoto.

Image orientation was done using physical points on the structure whose coordinates were extracted manually on the laser scans, as previous tests validated this operation. After calculation we verified that the average GSD of all the images was about 0.003 m. The mean error in camera alignment was about 1.8 pixels meters, after optimization. The rototranslation of the model for the georeferencing in the local reference system showed an error of about 0.011 m.

As result, dense cloud and mesh models were exported from Agisoft Photoscan. The first one was useful to integrate the scanner data after noise reduction and outliers removal. Mesh models were used to extract high-resolution orthophoto (Figure 8c) even if a healing process was necessary. This optimization was done using Rapidform XOR3.

To obtain a better orthophoto in terms of image quality (coherence of lights and shadows on the facades) not all images were used. Images with strong chromatic variations and especially non-parallel to the surface images were discarded. For the orthophotos of the bell tower facades we used fewer photographs: 125 images for the South front, 83 images for the north face, 69 images for the east, 99 images for the west (from the south-west front in clockwise). The round temple orthophoto was built with 154 images.

The first operation for the laser scanner data was alignment in the local reference system made by topography. The entire database of points acquired by laser scanner is divided into internal data (about 1.5 billion) and external ones (800 million only for the lower external part). The oriented point clouds were cleaned up from overlapping redundant data, noise points and outliers filtered away. The presence of a huge scaffold structure made this kind of work extremely time consuming because it was necessary to isolate the building point data from the scaffold data.

**Figure 9:**
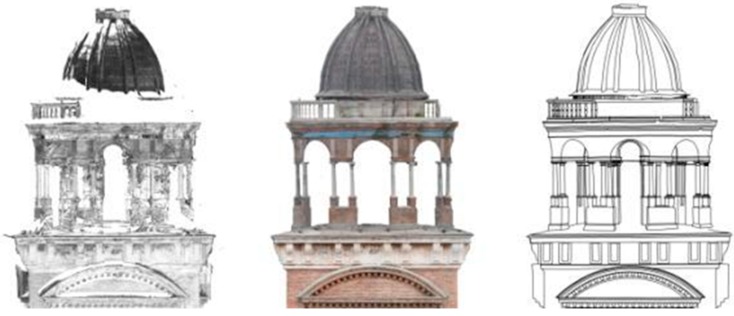
Comparison between the Santa Barbara bell tower’s top laser scanner data and the respective surface orthophoto elaborated from the photogrammetric 3D model.

**Figure 10:**
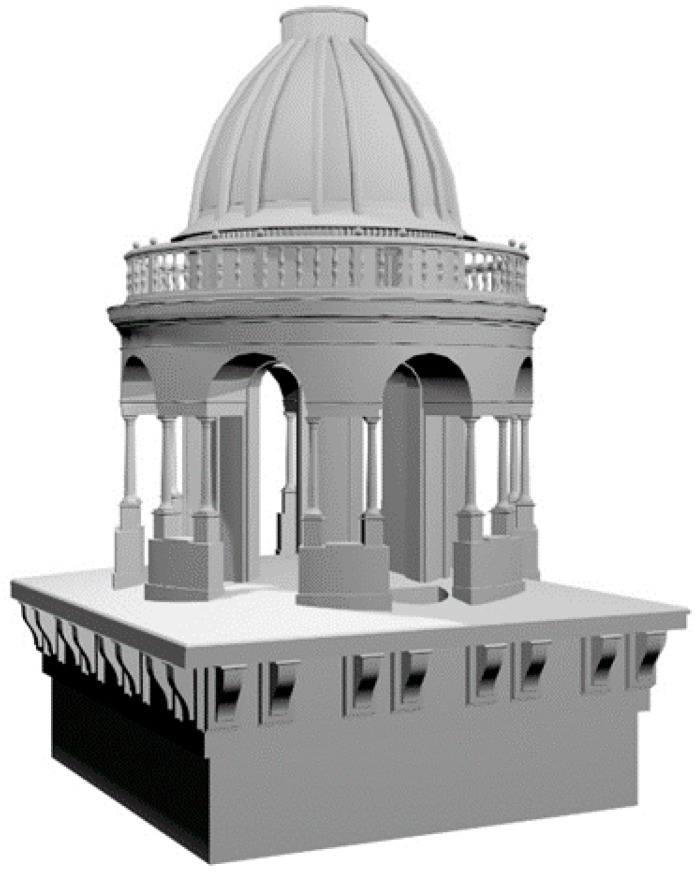
3D model of the round temple for FEM analysis.

**Figure 11:**
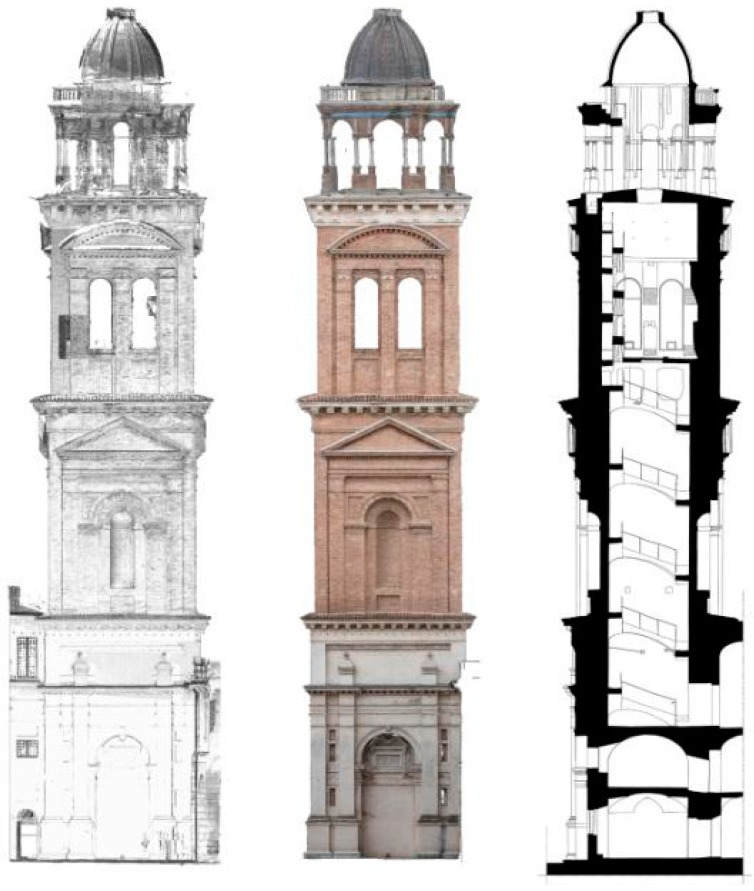
Santa Barbara bell tower. Laser scanning and photogrammetry data and a final elaboration of a vertical section obtained using both the technologies.

Point clouds and orthophotos were used together to realize the documentation of the actual post-earthquake situation [9]. According to the Cultural Heritage office of Mantua Diocese, some traditional architectural representations were drawn at a scale of 1:50: a plant for each tower floor with three additional horizontal sections in correspondence to the most damaged parts, two vertical sections of the complete structure and fronts integrated with orthophotos (Figure 9, Figure 10 and Figure 11).

Laser scanner point clouds were used to draw the plants at different floors and the interior part of the sections. According to the known pipeline, point clouds were imported in AutoCad and used as the basis for the drawings. The remaining part of the sections and the exteriors façades were drawn by using both laser scanner and photogrammetric point clouds. After bell tower restitutions the front’s drawings were overlapped with orthophotos to integrate vector information with color data.

For the round temple and the lantern, the most damaged parts of the belfry, in addition to the 3D drawings (Figure 9) the Diocese also asked for a 3D model. The project of restoration and seismic retrofitting was very complex, therefore it was necessary to provide more information. The construction engineers needed a 3D model for FEM analysis.

The 3D model was built by using laser scanner and photogrammetric data together. According to the requirements of structural engineers, the 3D model was built as a solid model using simple geometric primitives modified with Boolean operations (Figure 10).

## 7. Conclusions

This paper focuses on the survey of vertical structures in dense urban contexts in the case of emergency conditions. This experience proves that UAV acquisition and automated 3D image modeling are an effective way to achieve fast and precise results. To reach good results a correct flight project for the UAV acquisition is fundamental, in order to obtain a photogrammetric model with good image overlap and the optimal number of images to acquire all the architecture information avoiding overabundances. This technology is cheaper than TLS surveys thanks to the use of non-metric cameras and the use of automated software for the elaboration process, which minimizes human intervention. Above all, this approach allows the problem of surveying tall buildings in old town centers or congested areas to be solved effectively.

This methodology is very promising also because it can be fully integrated with other kinds of technology and instruments that proved their great efficiency in other similar cases of emergency (TLS and topography). The results of accuracy tests allow data from UAV automated photogrammetry to be fully integrated with laser scanner point clouds. From the database, based on all data, many representations can be extracted, both vectorial (plans, sections) and raster (facade orthophoto).

This integration of sensors ensures not only the quality of results, but also a safe working environment for the surveyor, even in post-earthquake cases. This operational methodology is fully integratable into the architectural process of damage assessment and planning of restoration or maintenance.
